# P-77. Ceftaroline versus Daptomycin as Definitive Treatment for Methicillin-Resistant *Staphylococcus aureus* Bone and Joint Infections

**DOI:** 10.1093/ofid/ofae631.284

**Published:** 2025-01-29

**Authors:** Reid Goodman, Katheryn Ney, Stephen Y Liang, Jonas Marschall

**Affiliations:** Washington University School of Medicine, St. Louis, Missouri; Washington University School of Medicine, St. Louis, Missouri; Washington University School of Medicine, St. Louis, Missouri; Washington University School of Medicine in St. Louis, Saint Louis, Missouri

## Abstract

**Background:**

There are no comparative effectiveness studies of ceftaroline versus other antibiotics for MRSA bone and joint infections (BJI). We retrospectively compared the clinical outcomes of patients with MRSA BJI initially treated with vancomycin and then transitioned to either ceftaroline (VtoC) or daptomycin (VtoD).

**Methods:**

This retrospective nested case-control study analyzed data from a large Midwest academic center collected from 2018-2022. Patients with MRSA BJI whose definitive treatment was ceftaroline were compared in a 1:2 ratio to patients whose definitive treatment was daptomycin. The primary outcome was clinical success after initial treatment course (i.e., at 6 weeks) defined by a composite endpoint which included resolution of signs and symptoms and no repeat surgery or re-admission for treatment of the index BJI.

**Results:**

We identified 36 patients to be included in this case-control study, with 12 VtoC cases and 24 VtoD cases. The demographic make-up was similar, as was the frequency of comorbidities (including Charlson score). Of the VtoC patients, 42% had implant-related infection vs. 17% of the VtoD group (p=0.1). A larger proportion of VtoC patients had systemic MRSA infection as documented by positive blood cultures (58% vs 38%; p=0.2). More VtoC cases were direct ICU admits (50% vs 13%; p=0.04), required intubation (58% vs 17%; p=0.02), or were admitted to the ICU at any point during their stay (67% vs 29%; p=0.07). Transition away from vancomycin occurred at 14.3 vs 10.6 days (p=0.6). There was only a single clinical success in the 12 VtoC patients (8%) vs. 7/24 (29%) in the VtoD group (p=0.2), with the majority of cases being failures (30.5%), all-cause deaths (30.5%), or lost to follow-up (16.7%). Follow-up was 103 vs 123 days (p=0.8) in the two groups.

**Conclusion:**

In this comparison of ceftaroline to daptomycin for MRSA BJI we found ceftaroline recipients to have more severe illness (as suggested by need for ICU care) and experience very little clinical success. We suspect that there is selection bias in that more severely ill cases anticipated to have poor outcomes were more likely to be transitioned to ceftaroline. Our next steps are to include more data and conduct a propensity score analysis to better understand what factors are associated with transition to ceftaroline.

**Disclosures:**

**All Authors**: No reported disclosures

